# A whole genome sequence association study of muscle fiber traits in a White Duroc×Erhualian F_2_ resource population

**DOI:** 10.5713/ajas.18.0767

**Published:** 2019-07-01

**Authors:** Tianfu Guo, Jun Gao, Bin Yang, Guorong Yan, Shijun Xiao, Zhiyan Zhang, Lusheng Huang

**Affiliations:** 1 State Key Laboratory of Pig Genetic Improvement and Production Technology, Jiangxi Agricultural University, Nanchang 330045, China; 2 Department of Biochemistry and Molecular Biology, Gannan Medical University, Ganzhou, Jiangxi 341000, China

**Keywords:** Pig, Imputation, Haplotype, Muscle Fiber

## Abstract

**Objective:**

Muscle fiber types, numbers and area are crucial aspects associated with meat production and quality. However, there are few studies of pig muscle fibre traits in terms of the detection power, false discovery rate and confidence interval precision of whole-genome quantitative trait loci (QTL). We had previously performed genome scanning for muscle fibre traits using 183 microsatellites and detected 8 significant QTLs in a White Duroc× Erhualian F_2_ population. The confidence intervals of these QTLs ranged between 11 and 127 centimorgan (cM), which contained hundreds of genes and hampered the identification of QTLs. A whole-genome sequence imputation of the population was used for fine mapping in this study.

**Methods:**

A whole-genome sequences association study was performed in the F_2_ population. Genotyping was performed for 1,020 individuals (19 F_0_, 68 F_1_, and 933 F_2_). The whole-genome variants were imputed and 21,624,800 single nucleotide polymorphisms (SNPs) were identified and examined for associations to 11 *longissimus dorsi* muscle fiber traits.

**Results:**

A total of 3,201 significant SNPs comprising 7 novel QTLs showing associations with the relative area of fiber type I (I_RA), the fiber number per square centimeter (FN) and the total fiber number (TFN). Moreover, one QTL on pig chromosome 14 was found to affect both FN and TFN. Furthermore, four plausible candidate genes associated with FN (kinase non-catalytic C-lobe domain containing [*KNDC1*]), TFN (*KNDC1*), and I_RA (solute carrier family 36 member 4, contactin associated protein like 5, and glutamate metabotropic receptor 8) were identified.

**Conclusion:**

An efficient and powerful imputation-based association approach was utilized to identify genes potentially associated with muscle fiber traits. These identified genes and SNPs could be explored to improve meat production and quality via marker-assisted selection in pigs.

## INTRODUCTION

Pork serves as one of the main animal protein sources in human diets, with its basic composition unit being the muscle fiber. With the rapid increases in living standards, consumers’ demand for higher quality meat has been increasing. One of the most important factors that influence meat/muscle quality is its histological characteristics. There are three main muscle fiber types in swine: slow-twitch type I, fast-twitch type IIA, and IIB [1]. The area, relative area and percentage of each fiber type are crucial to pork quality, affecting its color, ultimate pH, drip loss, tenderness and water-holding capacity etc. Muscle with higher proportion of type I fibers is more tender and favorable for meat quality [2], while a higher type IIB content tends to cause a pale, soft and exudative meat [3,4]. Fiber numbers and area are positively correlated with a lean meat percentage and muscle mass [5].

Genetic studies of muscle fiber traits are limited. A total of 113 quantitative trait loci (QTLs) for swine muscle fiber traits have been reported in the AnimalQTLdb database up to now [6]. Fifteen significant QTLs were associated with muscle fiber traits in a Japanese wild boar ×Large White population [7]. Thirteen QTLs for muscle fiber composition were identified in a Duroc×Berlin Miniature cross population [8]. Estellé et al [9] detected 20 significant genome regions associated with muscle fiber traits in an Iberian× Landrace F_2_ pig population. We have previously evidenced 8 genome-wide significant QTLs for muscle fiber traits in a Chinese Erhualian×White Duroc F_2_ resource population [10]. The confidence intervals of most QTLs were more than 10 centimorgan (cM) and massive numbers of genes were present in these regions, which hampered the further fine mapping and characterization of potential candidate genes.

With the development of high-throughput genotyping technology, genome-wide association studies (GWAS) have been extensively conducted for traits of interest in farm animals. To our knowledge, few GWASs for swine muscle fiber traits have been reported. Guo et al [11] performed a genome-wide association study to detect QTLs for muscle fiber and eye muscle traits in a Large White×Min pig F_2_ resource population. Twenty-four single nucleotide polymorphisms (SNPs) were identified to be associated with 6 muscle fiber traits, including 1 significant SNP associated with the red muscle fiber rate located on pig chromosome (Sus scrofa, SSC) 6; 4 significant SNPs associated with the white fiber rate on SSC7 and SSC11; 2 significant SNPs associated with the intermediate fiber rate on SSC14; 7 significant SNPs associated with muscle fiber area on SSC3, SSC4, and SSC9; 5 significant SNPs associated with muscle fiber density on SSC6; and 5 significant SNPs associated with muscle fiber diameter on SSC3 and SSC9 [11]. Li [12] performed GWAS for fiber traits in Large White×Min pig F_2_ resource population using a mixed linear model and detected 37 significant SNPs to be associated with muscle fiber diameter, muscle fiber area, and muscle fiber density, which were located on SSC3, SSC4, SSC5, SSC6, SSC7, and SSC14. GWAS partly overcomes the inadequacy of QTL mapping strategy, but repeatability is poor and confidence intervals are still too large to search for candidate genes.

To fine map and overcome the weakness of detecting small-effect QTLs using a low-density BeadChip, we sequenced 19 founders of the F_2_ resource population and 98 unrelated individuals (4 Erhualian pigs, 6 Wuzhishan pigs, 6 wild boars, 6 Luchuan pigs, 46 Tibetan pigs, 6 Hetao pigs, 6 Laiwu pigs, 6 Jinhua pigs, 6 Min pigs, and 6 Bamaxiang pigs) and reconstructed their haplotypes as a reference haplotypes library. The whole-genome sequence variants of the F_2_ individuals were then imputed from the reference haplotype library, and were used to test their associations with 11 muscle fiber traits, including numerical percentage; area and relative area of type I, IIA, and IIB myofibers; fiber number per square centimeter (FN); and total fiber number (TFN). These identified significant loci could aid in the genetic improvements of meat quality via marker assisted selection in swine breeding programs.

## MATERIALS AND METHODS

### Animals and phenotype measurements

All procedures involving animals followed the guidelines for the care and use of experimental animals (GB/T 27416-2014, Laboratory animal institutions-general requirements for quality and competence) approved by the National Standard of the People’s Republic of China. The ethics committee of Jiangxi Agriculture University specially approved this study.

A three-generation White Duroc×Erhualian F _2_ resource population was established as described previously [13]. In short, two White Duroc boars were mated to 17 Erhualian sows as founders. Nine F_1_ boars and 59 F_1_ sows were then randomly chosen and mated to produce a total of 1,912 F_2_ individuals in six batches. All F_2_ piglets were raised under the same conditions at a pig farm in Jiangxi Agricultural University (China). Animals were slaughtered at the age of 240±3 days at a slaughter facility following industry procedures. Herein, 11 fiber traits within the *longissimus dorsi* muscle were measured on 120 F_2_ animals that were divided into two batches. These 11 traits included area (A), relative area (RA), and numerical percentage (NP) of the three types of myofibers (I, IIA, and IIB) and FN and FTN. This approach was based on previous study that measured muscle fiber traits [1,10].

### DNA collection and genotyping

Genomic DNA was extracted from swine ear tissues using a standard phenol–chloroform method. The DNA samples were then quantified using a Nanodrop 1,000 spectrophotometer (Thermo Scientific, Waltham, MA, USA) and diluted to a concentration of 50 ng/μL in a 96-well plate as previously described [13]. A total of 1,020 individuals (19 F_0_, 68 F_1_, and 933 F_2_) were genotyped using the Illumina PorcineSNP60 BeadChip (62,163 SNPs) according to the manufacturer’s protocols. The genotyping data was then filtered by a call rate of no less than 0.95 and a Mendel error of no more than 0.05.

In this study, a whole-genome sequence imputation of the F_2_ population was carried out using IMPUTE2 tools with a basic scenario of a one-phased reference panel [14]. In order to obtain more accurate phases of the 19 F_0_, 98 unrelated individuals were added into the reference panel. Briefly, 117 pigs (19 F_0_ and 98 unrelated individuals) were re-sequenced using an Illumina Hiseq 2000, with an average depth of ~25 coverages being regarded as a reference panel. SNP calling was performed using Genome Analysis Toolkit (GATK, version 3.7) tools, with individual genotypes being generated using the GATK Haplotypecaller as previously described [15]. The raw whole genome SNPs were filtered using GATK genotype GVCFs and variant filtration options as previously described [16]. Only SNPs with a quality depth >5, mapping quality >30, Fisher Strand >60, and haplotype score >13 were kept for further analysis. The sequence data were then filtered, with samples of a minor allele frequency (MAF) >0.01 being retained. The haplotypes of the reference panel were inferred using SHAPEIT2 [17]. Whole genome sequences of F_2_ individuals were imputed using IMPUTE2 with default options (version 2.3.2) and the imputation accuracy was estimated using an internal cross-validation strategy. A cutoff of 0.3 was set for post-imputation SNP filtering and the clean data was converted to a BIMBAM file using a bash script in the GEMMA software manually [18]. Finally, SNPs with a MAF <0.03 were excluded.

### Single-trait genome-wide association studies analysis

Whole genome sequence associations were built based on a linear mixed model and implemented in the GEMMA software (version 0.94), which is described in the following equation:

$$\begin{array}{l} y=W\alpha +x\beta +u+ɛ\\ u\sim {\text{MVN}}_{n}(0,\lambda \tau^{-1}K),\;\;\;ɛ\sim {\text{MVN}}_{n}(0,\tau^{-1}I_{n}) \end{array}$$

where **y** is the vector of phenotypic observation; **W** is a matrix including covariates and a column of 1s; ***α*** is a vector of fixed effects (e.g., gender); **x** is a vector of genotypes; ***β*** is the effect size of the marker; **u** is a vector of random effects following the multivariate normal distribution; τ^−1^ represent the variance of the residual errors and λτ^−1^ represent the variance of the random effect, λ is the ratio between the two variance of the residual errors; **K** is a kinship matrix that is estimated from the whole genome sequence variants and is calculated following the formula:

$$\frac{(M-2P)\;(M-2P{)}^{\prime }}{p_{i}q_{i}}$$

ɛ is a vector of errors following the multivariate normal distribution, with **I***_n_* being an identity matrix. With high density markers throughout the whole genome, a naïve Bonferroni correction of 0.05 divided by the examined SNP number was used and generated an overly conservative threshold due to the SNPs being highly correlated with each other; thus deviating from the assumption of independent tests when performing Bonferroni corrections. We herein took the empirical distribution of p values of whole genome markers and calculated the genome-wide false discover rate (FDR) following Storey [19] and Benjamini and Yekutieli [20]. The mathematic formula expression of the FDR as:

$$\text{FDR }({\text{P}}_{i})={\text{P}}_{i}\times \frac{m}{order(p_{i}),}$$

where m is the largest p value, P*_i_* is p value of tested SNP and *order*(*p_i_*) is the smallest p value. Herein, we assumed that independent haplotype segments between pigs and humans are equal, thus the same genome-wide threshold was utilized. The 95% confidence interval for each QTL was determined by finding the regions on both sides of a given candidate SNP that correspond to a decrease of −2 log (p-value) units according to the study of Rebai et al [21].

### Candidate genes selection

Candidate genes were carefully inspected within one Mb area around candidate SNPs using the Ensembl browser (http://ensembl.org/Sus_scrofa/Info/Index) and Gene Cards (http://www.genecards.org) were used to determine the functions of the genes.

## RESULTS

### Phenotype statistics

**Figure 1:**
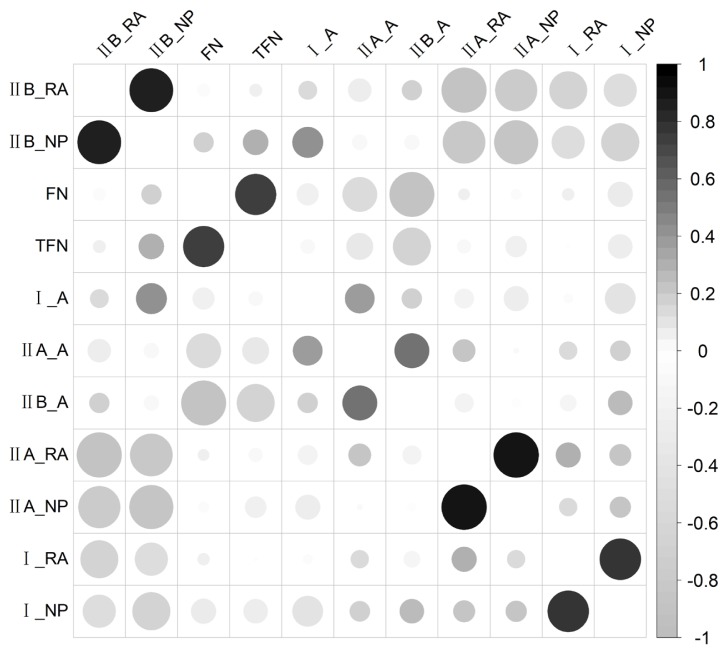
Correlations among muscle fiber traits. The positive and nagtive correlations are exhibited in black and gray respectively, the size of the circles and degree of the shadow represent the strength of the correlations, the larger circles and deeper shadow represent stronger correlation.

One hundred and twenty F_2_ individuals were measured for muscle fiber traits, of which 100 individuals were genotyped and analyzed and detailed descriptive statistics of the traits were presented in our previous study [10]. The relative area of fiber type IIB (IIB_RA) and the numerical percentage of fiber type IIB (IIB_NP) were highly negatively correlated with the relative area of fiber type IIA (IIA_RA), the numerical percentage of fiber type IIA (II A_NP), the relative area of fiber type I (I_RA) and the numerical percentage of fiber type I (I_NP; Figure 1). The FN and the TFN were moderately negatively correlated with the areas of fiber types IIA (IIA_A) and IIB (IIB_A). Furthermore, FN, I_NP, IIA_NP, and IIB_NP were highly positively correlated with TFN, I_RA, IIA_RA, and IIB_RA, respectively (Figure 1).

### Imputation summary

A total of 21,624,800 SNPs across the whole genome were imputed using IMPUTE2, which ranged between 608,266 and 2,038,996 across chromosomes. Post-imputation SNP filtering was then performed using the information metric with a cutoff of 0.3 and 6,434,337 SNPs were eliminated. This resulted in 15,190,563 SNPs being retained (Table 1), while 441,113 SNPs were removed due to a low imputation accuracy. The imputation accuracy varied from 91% to 96% with an average accuracy of 94%.

### Single-trait genome-wide association studies

**Figure 2:**
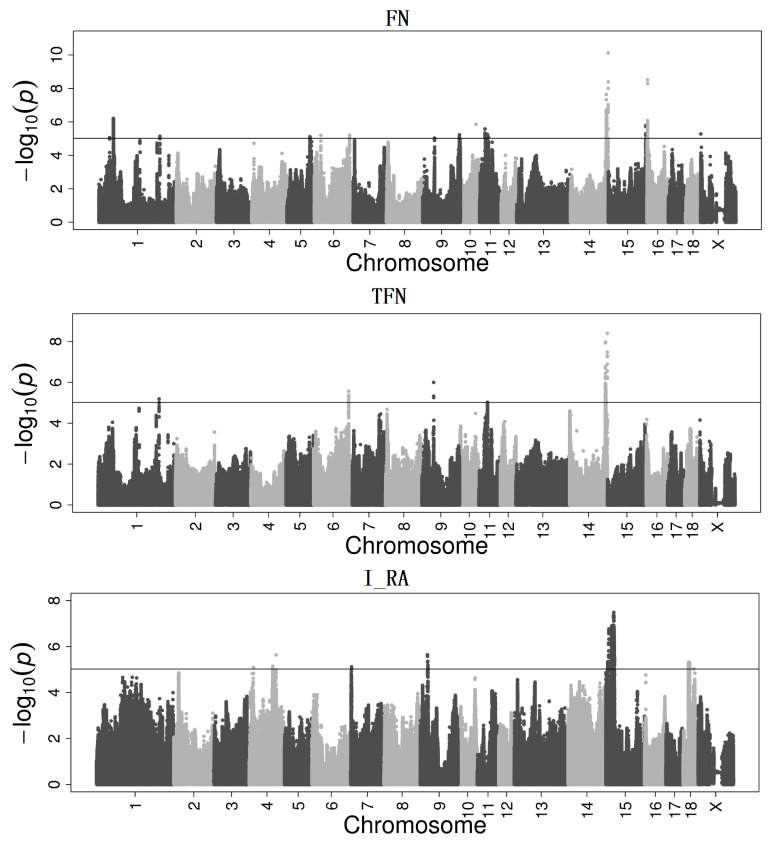
The related Manhattan plots of GWAS results for muscle fiber traits. The x-axis and y-axis respectively represent the genomic positions separated by chromosomes and the –log10 (p-value) of the SNPs, different chromosomes are separated by gray and black colors, the black solid lines indicate the chromosome significance threshold. GWAS, genome-wide association studies; SNPs, single nucleotide polymorphisms; FN, fiber number per square centimeter; TFN, total fiber number; I_RA, relative area of fiber type I.

In total, 3,201 genome-wide significant SNPs associated with 3 muscle fiber traits and located on SSC4, SSC7, SSC9, SSC14, SSC15, SSC16, and SSC18 were identified. Among these SNPs, the majority SNPs are associated with I_RA (3,192 SNPs) located on SSC4, SSC7, SSC9, SSC15, and SSC18; and the rests are associated with TFN (3) and FN (6) located on SSC14 and SSC16. The substitution effects and the frequency of top SNP for each QTL was listed in Table 2 and whole genome association profiles for the three traits were exhibited by the Manhattan plots (Figure 2).

For each QTL, in order to verify whether other detected significant SNPs are the same QTL as the top SNP, we took the genotype of the top SNP as covariant variable contained in the mix model frame and carried out conditional GWAS analysis again. The result showed that the original QTL signal disappeared and no new QTL loci appeared, indicating that these SNPs are controlled by the same QTL.

Two significant QTLs associated with FN were identified. One QTL is located on SSC14 at position 153,137,973 bp (p = 7.52E-11) with a confidence interval of 154.64 kb and substitution effect of 2.72E+04 (on average FN for individuals carried TT genotype is 2.72E+04 more than that for individuals carried CT genotype). This QTL was found to be located within the intron of the kinase non-catalytic C-lobe domain containing (*KNDC1*) gene. The other one is located on SSC16 at position 2,353,144 bp (p = 3.04E-09), the confidence interval is 1.36 kb, with only two SNPs exceeding the significance threshold, and a substitution effect of 2.10E+04. The most closely associated gene for this QTL was *ENSSSSCG000000 29792* and its function is currently unknown. Furthermore, for TFN, the same results for FN on SSC14 were observed. For I_RA, 5 QTLs located on SSC4, SSC7, SSC9, SSC15, and SSC18 were identified at position of 109,585,219, 3,489,379, 28,986,217, 31,876,859, and 23,539,146 bp, respectively. The most closely associated genes for these QTLs are *ENSSSSCG 00000024957*, *ENSSSCG00000001016*, solute carrier family 36 member 4 (*SLC36A4*), the contactin associated protein-like 5 (*CNTNAP5*), and glutamate metabotropic receptor 8 (*GRM8*). For the remaining muscle fiber traits, no significant signals were observed (FDR>0.05).

**Figure 3:**
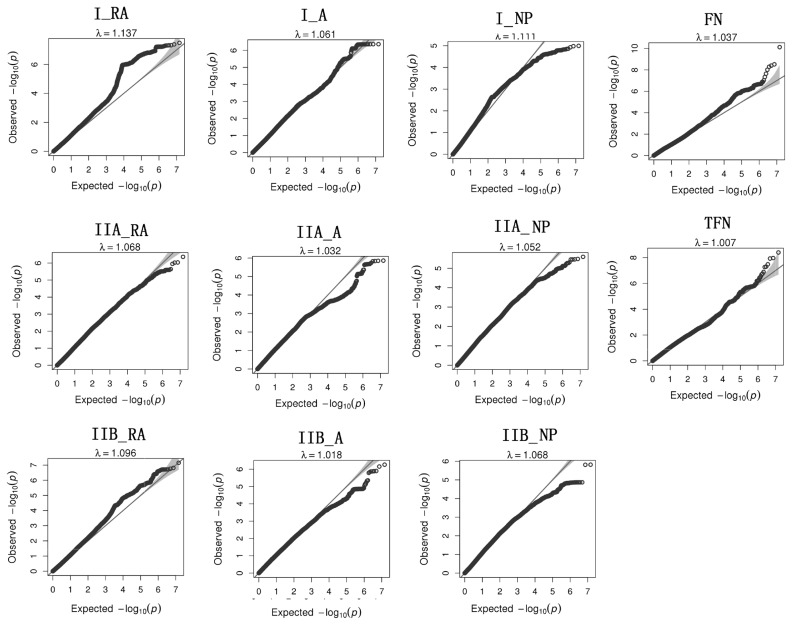
Q-Q plots for assessing the influence of population stratification. The x-axis and y-axis represent the expected and observed –log10 (p-values), respectively.

In general, GWAS can easily generate false positive signals in F_2_ populations if population stratifications are not properly corrected [22]. To confirm that the population stratification was properly adjusted herein, quantile-quantile plots were constructed for each trait (Figure 3). The genome inflation coefficient (λ) ranged between 1.007 and 1.110, thus indicating that population stratification has a negligible effect on the results.

## DISCUSSION

The proportion of muscle fiber types and their area are important economic traits related to meat quality. With the rapid development of high throughput and cost-effective whole genome genotyping techniques, the patterns of genetic variation and their association with swine muscle fiber phenotypes can be examined. Collecting muscle fiber type phenotypic data is a very laborious and complicated process; thus only 120 out of 1,912 F_2_ individuals were phenotyped. Until now, little research has been done regarding the identification of QTLs or SNPs associated with these traits [7–9,11,12]. In our previous study, the following QTLs were identified within the same sample population: 3 QTLs associated with IIB_A on SSC7, SSC8, and SSC15; 3 QTLs associated with IIA_A on SS7 and SSC11; 1 QTL associated with FN on SSC7; and 1 QTL associated with I_NP on SSCX. The confidence interval for these QTLs ranged between 11 and 127 cM [10]. Interestingly, fewer QTLs and no consistent QTLs were detected in the present GWAS study compared with previous studies.

A QTL scan assumes that Q or q are alternatively fixed in one founder line, with phenotype segregation occurring due to the recombination of the two lines during meiosis. Linkage analysis seeks to detect long chromosomal segments co-segregating with QTL genotypes and is designed to identify large-effect QTLs with large confidence intervals. However, GWAS is designed to detect moderate- or small-effect QTLs with a relatively small confidence interval. GWAS assumes that the analyzed marker is in high linkage disequilibrium with a causative mutation, possibly attributing to the difference in results. Additionally, the threshold used in linkage mapping and GWAS differ, with a stringent correction threshold used in GWAS which may decrease the detection power.

In the present study, if two significant SNPs were identified on the same chromosome and the distance between these two SNPs is less than 5 Mb, they are treated as identical QTL. Based on this simple rules, 3,201 significant SNPs comprising 7 QTLs were identified. Moreover, one QTL on SSC14 was found to be associated with both FN and TFN, thus indicating a multi-effect QTL. Genes closest to these significant SNPs included *KNDC1* (one significant SNP is located in this gene), *KCNA2* (one significant SNP is located in this gene), *SLC36A4*, *GRM8 CNTNAP5*, *ENSSSCG00000024957*, *ENSSS CG00000001016* (one significant SNP is located in this gene) and *ENSSSSCG00000029792*. However, in terms of functional annotation, these genes are not directly related to muscle fiber traits. We herein extended gene searching to ~ 1-Mb region near to these SNPs, just a disintegrin and metalloproteinase domain-containing protein 8 (*ADAM8*) gene and bridging integrator-1 (*BIN1*) gene stand out for QTLs on SSC14 and SSC15 respectively.

*ADAM8* is a member of the disintegrin and metalloprotease (*ADAM*) family and is located on SSC14 with a confidence interval of 154.64 kb. *ADAM8* inhibition is characterized by increased muscle degeneration and increased numbers of necrotic and calcified muscle fibers. *ADAM8* enhances neutrophils invasiveness into injured muscle fibers by reducing their adhesiveness to blood vessels after infiltration into interstitial tissues [23]. *ADAM8* is also the primary protease for α-cleavage of cellular prion protein and appears to be self-regulated in muscle tissue [24].

The *BIN1* gene was found to be located near the QTL on SSC15, and its expression, structure and localization have been reported to be tightly regulated during muscle differentiation [25]. *BIN1* plays a critical role in cardiac muscle development [26]. Johann et al [27] demonstrated that the alteration of the muscle-specific function of amphiphysin 2 (encode by *BIN1*) was a common pathomechanism for centronuclear myopathy, myotonic dystrophy, and inherited myopathy in Great Danes. Furthermore, reproductive *BIN1* splicing alterations has been shown to promote T-tubule alterations and muscle weakness [28].

The family with sequence similarity 105, member A (*FAM105A*) gene, which is located 1.977 Mb from the identified SNP on SSC16, has been shown to play a role in apoptosis, but its overall biological function is still unclear. Overexpression of *FAM105A* was found to increase levels of the pro-apoptotic factor *Bax*, while decreasing levels of the anti-apoptotic factor *Bcl-2* [29]. It is possible that *FAM105A* promotes myofibroblast apoptosis, thereby affecting muscle fiber numbers.

## CONCLUSION

In this study, GWAS was performed to detect QTLs for muscle fiber traits in a F_2_ resource population. Imputed whole genome sequence data was obtained and 3,201 significant SNPs showed associations with I_RA, FN and TFN, corresponding to 7 novel QTLs for these traits on SSC4, SSC7, SSC9, SSC14, SSC15, SSC16, and SSC18. The most plausible candidate genes at the identified loci included *ADAM8*, *BIN1*, and *FAM105A*.

## Figures and Tables

**Table 1:** The distribution of SNPs in different chromosomes

Chr	Total_variants1)	Cutoff 0.32)	MAF 0.033)
1	2,038,996	1,429,949	1,387,709
2	1,435,251	917,703	890,350
3	1,264,324	833,350	812,554
4	1,198,042	948,333	922,849
5	1,016,156	747,823	726,892
6	1,311,375	921,892	899,681
7	1,240,292	928,263	894,310
8	1,291,810	872,534	850,403
9	1,340,821	980,268	938,963
10	1,010,012	698,331	683,259
11	885,878	661,072	643,340
12	661,658	497,369	483,160
13	1,478,410	963,155	934,979
14	1,248,247	1,002,735	967,714
15	1,196,998	816,687	791,339
16	814,627	596,531	577,766
17	693,090	527,157	507,938
18	608,266	413,003	404,906
X	890,547	434,408	431,338
Total	21,624,800	15,190,563	14,749,450

SNPs, single nucleotide polymorphisms; Chr: chromosome number; MAF, minor allele frequency.

1) Total SNPs.

2) Number of SNPs remained after filtering with post-imputation SNP information metric of 0.3.

3) Number of SNPs remained after filtering with a MAF of 0.03.

**Table 2:** Description of SNP significantly associated with muscle fiber traits

Trait	Chr1)	Pos (bp)2)	number of SNPs	REF (Allele)	ALT (Allele)	Freq3) (REF)	FDR value	Beta4)	Nearest gene5)	Dis (bp)6)
TFN (/cell)	14	153,137,973	3	C	T	0.768	0.05	−1.05E+06	*KNDC1*	within
FN (/cm^2^)	14	153,137,973	4	C	T	0.768	0.01	−2.72E+04	*KNDC1*	within
	16	2,353,144	2	G	A	0.133	0.05	2.10E+04	*ENSSSSCG00000029792*	1,483,087
I_RA (%)	4	109,585,219	10	A	G	0.51	0.05	−1.91E-02	*ENSSSSCG00000024957*	43,437
	7	3,489,379	3	T	C	0.962	0.05	−4.44E-02	*ENSSSCG00000001016*	within
	9	28,986,217	29	A	G	0.941	0.05	−4.44E-02	*SLC36A4*	71
	15	31,876,859	2,962	G	T	0.061	0.01	3.96E-02	*CNTNAP5*	69,863
	18	23,539,146	188	C	G	0.961	0.05	−8.58E-02	*GRM8*	447,205

SNP, single nucleotide polymorphism; REF, reference; ALT, alternate; FDR, false discover rate; TFN, muscle fiber traits, the total fibers number of a longissimus dorsi muscle; *KNDC1*, kinase non-catalytic C-lobe domain containing; FN, the fiber number per square centimeter; I_RA, relative area of fiber type I; *SLC36A4*, solute carrier family 36 member 4; *CNTNAP5*, contactin associated protein-like 5; *GRM8*, glutamate metabotropic receptor 8.

1) Chromosomal locations of the significant SNPs.

2) Positions of the significant SNPs according to *sus scrofa* 10.2 genome assembly.

3) The frequency of reference allele of this population.

4) The substitution effect of the studied trait, which is the effect of reference allele substituted by the alternative allele.

5) The nearest genes from the significant SNPs.

6) The distance from the significant SNPs to the nearest genes.
